# Reduced virulence in tigecycline-resistant *Klebsiella pneumoniae* caused by overexpression of *ompR* and down-regulation of *ompK35*

**DOI:** 10.1186/s12929-023-00910-w

**Published:** 2023-03-31

**Authors:** Suyeon Park, Hyunkeun Kim, Kwan Soo Ko

**Affiliations:** 1 Department of Microbiology, Sungkyunkwan University School of Medicine, Suwon, 16419 Republic of Korea; 2 Department of Advanced Bioconvergence Product, Ministry of Food and Drug Safety, Cheongju, 28159 Republic of Korea

**Keywords:** *Klebsiella pneumoniae*, Tigecycline, *ompK35*, *ompR*, Hypermucoviscosity

## Abstract

**Background:**

The development of tigecycline resistance in hypervirulent *Klebsiella pneumoniae* strains has resulted in decreased virulence that is associated with reduced production of capsular polysaccharides (CPS). In this study, we investigated the mechanisms that link tigecycline susceptibility to decreased virulence.

**Methods:**

We compared transcriptomes from tigecycline-susceptible wild-type strains and tigecycline-resistant mutants using mRNA sequencing. *ompR*-overexpressed and *ompR*-deleted mutants were constructed from wild-type strains and tigecycline-resistant mutants, respectively. Antibiotic susceptibility tests were performed, and string tests and precipitation assays were conducted to identify phenotypic changes related to tigecycline susceptibility and *ompR* expression. Bacterial virulence was assessed by serum resistance and *Galleria mellonella* infection assays.

**Results:**

Transcriptomic analyses demonstrated a significant decrease in the expression of *ompK35* in the tigecycline-resistant mutants. We observed that tigecycline-resistant mutants overexpressed *ompR*, and that the expression of *ompK35* was regulated negatively by *ompR*. While tigecycline-resistant mutants and *ompR*-overexpressed mutants exhibited reduced hypermucoviscosity and virulence, deletion of *ompR* from tigecycline-resistant mutants restored their hypermucoviscosity and virulence.

**Conclusions:**

In hypervirulent *K. pneumoniae* strains, *ompR* expression, which is regulated by exposure to tigecycline, may affect the production of CPS, leading to bacterial virulence.

## Background

*Klebsiella pneumoniae* is one of the significant gram-negative pathogens that cause a variety of diseases including intra-abdominal infections, pneumonia, urinary tract infections, and pyogenic liver abscesses [1]. Several virulence factors, capsules, lipopolysaccharides, siderophores, and fimbriae have been identified in *K. pneumoniae* [2]. Particularly, the capsule is widely recognized as a major virulence factor that contributes to its defense against environmental pressures and host immune responses, as well as to antibiotic resistance [3, 4].

Due to the increased antimicrobial resistance in *K. pneumoniae*, tigecycline is often used as a last-resort antibiotic to combat multidrug-resistant *K. pneumoniae* [5]. However, tigecycline resistance has been reported with increased frequency in *K. pneumoniae* during treatments with tigecycline, or even without exposure to tigecycline [6, 7]. It has been known that resistance to tigecycline is mainly attributed to the overproduction of efflux pumps such as AcrAB and OqxA, or to mutations in efflux pump regulator genes such as *ramA*, *soxR*, *marR*, and *acrR* [8].

In gram-negative bacteria, the outer membrane proteins play a crucial role in bacterial virulence and are also associated with antibiotic resistance [9]. It is well-known that *K. pneumoniae* generates two major porins: OmpK35 and OmpK36, the levels of which are affected by a variety of environmental conditions such as osmolarity, temperature and pH [10]. The tigecycline-resistant *K. pneumoniae* isolates exhibited significantly decreased expression of the porin OmpK35, compared to susceptible isolates [11]. In addition, the development of tigecycline resistance in hypervirulent *K. pneumoniae* resulted in decreased virulence associated with reduced CPS [12]. However, it is not known why CPS and virulence decrease in the tigecycline-resistant *K. pneumoniae* strain.

In this study, we investigated the mechanism for the association between OmpK35 and tigecycline resistance. We constructed mutants with deleted or overexpressed *ompR*, a negative regulator of *ompK35*, and compared the mucoviscosity, virulence, and gene expression between wild-type *K. pneumoniae* strains and their *ompR* mutants.

## Materials and methods

### Bacterial strains, plasmids, and culture conditions

In this study, two *K. pneumoniae* strains, 109 and 200, were used that were isolated from the blood of South Korean patients [12]. Their capsular serotype was determined to be K1 and the 109 and 200 exhibited hypermucoviscous. Both strains belong to the multilocus sequence type 23 (ST23) and were susceptible to tigecycline. The tigecycline-resistant mutants 109-IR and 200-IR were derived from tigecycline-susceptible *K. pneumoniae* strains by methods previously described [12]. Briefly, the susceptible strains were subcultured in Luria–Bertani (LB) broth with a serially increasing concentration of tigecycline (0.5 to 64 mg/L). All *K. pneumoniae* strains, mutants and plasmids that were used to construct mutants are presented in Table 1. All bacterial strains were grown in LB broth with shaking at 37 °C, and tigecycline-resistant mutants were cultured in media with 64 mg/L tigecycline. Where appropriate, gentamicin (30 mg/L) was added to the growth medium, and isopropyl-β-d-thiogalactopyranoside (IPTG) was added to 109/ompR and 200/ompR at a concentration of 0.25 mM, and to 109-IR∆ompR-C and 200-IR∆ompR-C at a concentration of 1 mM to induce OmpR.

**Table 1:** Bacterial strains and plasmids used in this study

Strain or plasmid	Genotype or relevant characteristics	References
Strains		
SMC1204-109 (109)	*K. pneumoniae* ST23, Serotype K1, clinical isolate	[12]
SMC1207-200 (200)	*K. pneumoniae* ST23, Serotype K1, clinical isolate	[12]
SMC1204-109-IR (109-IR)	Tigecycline-resistant mutant developed from 109	[12]
SMC1207-200-IR (200-IR)	Tigecycline-resistant mutant developed from 200	[12]
109/ompR	109, *ompR*-overexpressed by IPTG	This study
200/ompR	200, *ompR*-overexpressed by IPTG	This study
109-IR∆ompR	109-IR, *ompR* inactivated	This study
200-IR∆ompR	200-IR, *ompR* inactivated	This study
109-IR∆ompR-C	109-IR∆ompR, *ompR*-complemented	This study
200-IR∆ompR-C	200-IR∆ompR, *ompR*-complemented	This study
Plasmids		
pUHE21-2*lacI*^q^	P_*lac*_ rep_pMBI_ Amp^R^ *lacI*^*q*^	[25]
pDK4	rep_R6Kγ_ Amp^R^ FRT Km^R^ FRT	[13]
pKD46	rep_pSC101_(Ts) Amp^R^ P_araBAD_ γ β exo	[13]
pCVD442	rep_R6Kγ_ Gm^R^	[26]
pHK251	pUHE21-2*lacI*^*q*^ with an insertion of Gm^R^ cassette from pCVD442	This study
pHK1009	pDK46 with an insertion of Km^R^ cassette from pKD4	This study
pHK1014	pKD46 with an insertion of Gm^R^ cassette from pJN105	[27]
pSY005	pHK251 containing *ompR*	This study

Amp^R^: ampicillin resistance; Km^R^: kanamycin resistance; Gm^R^: gentamicin resistance

### Transcriptomic analysis by mRNA sequencing

Transcriptome profiling was performed to contrast the expression profiles between tigecycline-susceptible and resistant strains. For mRNA sequencing, all isolates were overnight cultured with vigorous shaking (220 rpm) at 37 °C and diluted into fresh LB broth (1:100). The RNA samples were extracted from mid-log phase bacterial cultures using a Qiagen RNeasy Mini kit (Qiagen, Hilden, Germany), according to the manufacturer’s instruction. The TURBO DNA-free™ Kit (Invitrogen, MA, USA) was used to remove the contaminated DNA in RNA samples and we obtained mRNA from isolates. After isolation of RNA, cDNA was synthesized and sequencing libraries were generated in strand-specific manner according to the Illumine standard protocol for high-throughput sequencing. Library construction and sequencing were performed at Macrogen Inc. (Seoul, South Korea) using an HCS 3.3.52 Software for Illumina HiSeq 4000 sequencing system. The 101 bp paired-end raw reads were filtered and trimmed using Fase QC (version 0.11.7) and Trimmomatic (version 0.38). Expression levels of mRNA were measured as reads per kilobase per million sequence reads (RPKM), which considers the gene length for normalization. The complete genome sequence of *K. pneumoniae* NTUH-K2044 was used for aligning reads. The GenBank accession number of NTUH-K2044 is AP006725.

### Construction of *ompR* deletion mutants

The *ompR*-deleted mutants were generated from 109-IR and 200-IR using the Lambda-Red recombinase method [13] and the pKD46 plasmid (Table 1). The pHK1014 plasmid was introduced into 109-IR and 200-IR by electroporation. Transformants were selected using 30 mg/L gentamicin, and then bacterial colonies were confirmed using the primer pairs pKD46-repA101-F/pKD46-repA101-R (Table 2). The kanamycin resistance gene (Km^R^) in pHK1009 was amplified using the primer pairs Del-ompR-F/Del-ompR-R that are upstream and downstream to the ompR gene (Table 2). The Km^R^ amplicon was transformed into 109-IR and 200-IR which harbored pHK1014. Transformants were selected using 50 mg/L kanamycin, and bacterial colonies were confirmed using the primer pairs Checkpri-ompR-F/Checkpri-ompR-R (Table 2).

**Table 2:** Primers used in this study

Primers	Sequence (5′ → 3′)	Reference
Primers for cloning		
ompR-F/BamHI^a^	TGAGA**GGATCC**ATGCAAGAGAATTATAAGAT	This study
ompR-R/BamHI^a^	CTACA**GGATCC**TCATGCCTTAGAACCGTCCG	This study
ompR-F	GGTGATCAGCGGCGTTTTCAT	This study
ompR-R	AGTGTGCGAGCAAAGGAGCT	This study
Primers for deletion		This study
Del-ompR-F^b^	ACGCACTGACTATTGCAGTGAACCTTTGGGAGTACAAACATGTAGGCTGGAGCTGCTTCG	This study
Del-ompR-R^b^	GTGCGAGCAAAGGAGCTGCGCGGCGAAAAGCGCACGCGTTCATATGAATATCCTCCTTAG	This study
Primers for verification		This study
Checkpri-ompR-F	CAGTTTTTCATATCCCTCGCG	This study
Checkpri-ompR-R	CTGCAGTTTGTCGGTCATCA	This study
pKD46-repA101-F	CCCCACGATTGAAAACCCTACAAGG	This study
pKD46-repA101-R	GGAAAATCAACGTATCAGTCGGGCG	This study
Primers for qRT-PCR		This study
Q-ompR-F	ACCGAGCAGGGCTTCCA	This study
Q-ompR-R	CAGCATCAGATCCAGCACCAT	This study
Q-ompK35-F	TACGGCCAGTGGGAATACAAC	This study
Q-ompK35-R	CGTATTCGCCCGCTTTCA	This study

^a^Bold sequences refer to restriction enzyme cutting sites

^b^Underlined sequence refer to complementary to the plasmid sequence for overlapping PCR

### Cloning of *ompR* and complementation to mutants

For construction of the *ompR* expression vector, *ompR* was amplified from chromosomal DNA from the strain 109 using PCR and the primer pairs ompR-F/ompR-R (Table 2). The PCR product was purified using the extraction kit (iNtRON, Seongnam, Korea), digested with BamHI and ligated into a pHK251 precut using the same restriction enzyme. To confirm the mutation in *ompR*, Sanger DNA sequencing was performed. The pSY005 plasmid was electroporated into strains 109 and 200 for overexpression of *ompR* and it was also transformed into 109-IR∆ompR and 200-IR∆ompR for complementation by electroporation. The transformants were then plated on LB agar containing 30 mg/L gentamicin for selection.

### Antimicrobial susceptibility testing

Antimicrobial susceptibility testing was performed in accordance with the FDA guideline (https://www.accessdata.fda.gov/drugsatfda_docs/label/2013/021821s026s031lbl.pdf, susceptible, MIC ≤ 2 µg/mL; intermediate, MIC = 4 µg/mL; and resistant, MIC ≥ 8 µg/mL), since no tigecycline breakpoints exist in the Clinical Laboratory Standards Institute guideline for *K. pneumoniae*. The minimum inhibitory concentration (MIC) of tigecycline was determined by broth microdilution using *Escherichia coli* ATCC 25922 as the reference strain. All tests were performed in duplicate.

### String tests and precipitation assays

To conduct string tests, all strains were inoculated overnight on LB agar plates at 37 °C and bacterial colonies on plates were extended using a loop. Hypermucoviscosity was determined to be positive when the strain produced a stretched string more than 5 mm in length using a loop [12]. Precipitation assays were performed by centrifugation of the cultures. As the supernatant of hypermucoviscous strains remain dense after centrifugation, the supernatant density of centrifuged cultures can be a quantitative indicator of hypermucoviscosity [14]. Prior to centrifugation, all bacterial strains were cultured in LB broth at 37 °C overnight with shaking. The samples were centrifuged at 2000×*g* for 10 min then the bacteria were suspended and diluted until the optical density at 600 nm (OD_600_) reached 4. The optical densities (OD) of the supernatants were measured at OD_600_. All tests were performed three biological replicates per strain.

### Serum resistance assay

Serum resistance assays were performed to evaluate the resistance against killing by normal human serum (NHS) using a previously described method [12] with slight modifications. All strains were incubated overnight in LB broth at 37 °C with shaking and diluted 1:100 with fresh LB media and grown until the mid-log phase. Twenty-five μL of the bacterial solution were mixed with 75 μL of NHS in microtubes. Heat-inactivated human serum was used as a control to determine the ability to eliminate bacteria by NHS. The mixtures were incubated for 3 h with shaking and plated on LB agar after being serially diluted with phosphate buffered saline (PBS) for colony counting. For all strains, three independent tests were performed.

### Galleria mellonella infection assays

*Galleria mellonella* larvae were purchased from the Sworm Corp. (Cheonan, South Korea). The *G. mellonella* larvae were kept at room temperature in the dark with food for ten days before use. Larvae with weights of approximately 150–200 mg were selected for further experiments.

Bacterial infections of *G. mellonella* were performed as previously described with minor modifications [15]. Overnight bacterial cultures were harvested by centrifugation at 16,000 × *g* for 2 min then washed with 10 mM PBS. Bacterial cultures were adjusted with PBS to a McFarland standard of 0.5. The larvae were then infected with 10 μL of bacterial solutions by injection into the larvae’s last right proleg using an ultra-fine needle (BD Biosciences, San Jose, CA, USA). A PBS injection was used as a negative control, and *K. pneumoniae* strain ATCC 43816 as a positive control of hypermucoviscous strain. Ten larvae were infected with each bacterial strain and the viability of the larvae was examined until 72 h post infection. The experiments were performed three times independently.

### RNA extraction and quantitative RT-PCR

To measure the relative fold changes of expression of *ompK35* and *ompR* in the strains, quantitative real-time PCR (qRT-PCR) was performed. Overnight cultures of bacteria were inoculated into fresh LB broth and incubated at 37 °C with shaking until mid-log phase. Then, RNA was extracted using a Qiagen RNeasy Mini kit (Quiagen, Hilden, Germany) according to the manufacturer’s instructions. Contaminated DNA was eliminated from RNA samples using a TURBO DNA-free™ Kit (Invitrogen, MA, USA) and the reverse transcription reactions were conducted using a reverse transcription premix kit (iNtRON, Seongnam, South Korea). qRT-PCR was performed using TB Green Premix Ex Taq (TaKaRa, Shiga, Japan) with the QuantStudio 6 Flex Real-Time PCR system (Applied Biosystems, CA, USA) using the primers listed in Table 2. The expression levels were determined by the 2^−ΔΔCT^ method using the *rpoB* gene as a reference. The qRT-PCRs were performed three times and each sample was analyzed in duplicate.

### Statistical analysis

Statistical analyses of all experiments were performed to assess the significance of the differences using Student’s t-test, a one-way ANOVA with Tukey’s multiple comparisons test, and a nonparametric Kruskal–Wallis test followed by Dunnett’s multiple comparison test with Prism v8.3 for windows (GraphPad Software, San Diego, CA, USA). P values of < 0.05 were considered to be statistically significant (*, *p* < 0.05; **, *p* < 0.001, ***, *p* < 0.0001).

## Results

### Differentially expressed genes in the chromosome of *Klebsiella pneumoniae*

**Fig. 1:**
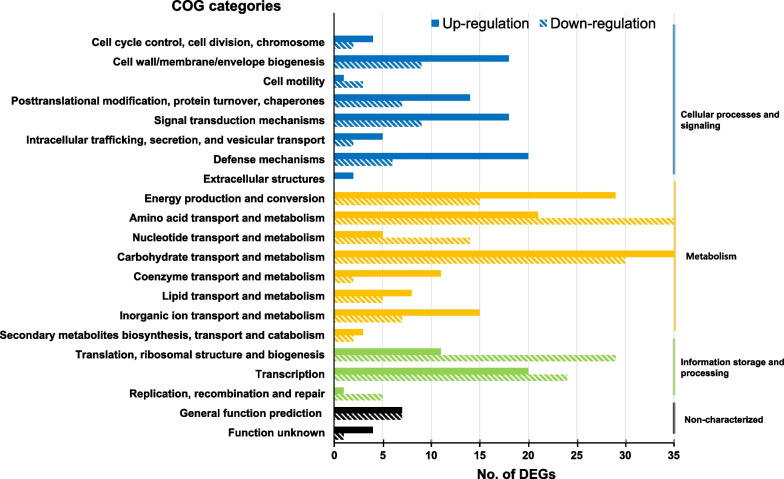
Results of transcriptome analysis. COGs of genes differently expressed in both tigecycline-resistant mutants, 109-IR and 200-IR, with criteria of fold-change ≥ 2. Solid bars indicate over-expressed genes and hashed bars indicate under-expressed genes

The chromosomal genes differently expressed in both tigecycline-resistant mutants (109-IR and 200-IR) were filtered with the criteria of fold-change ≥ 2. The filtered genes were categorized on the basis of the classification of clusters of orthologous groups (COGs) (Fig. 1). As a whole, 252 genes were up-regulated and 214 genes were down-regulated both in tigecycline-resistant mutants compared with the wild-type strains. Especially, 127 genes of metabolism pathway including carbohydrate transport and energy production were overexpressed.

Table 3 lists the top 10 genes with the highest differentially expressed genes in the tigecycline-resistant mutants compared with wild-type and tigecycline-susceptible *K. pneumoniae* strains. From the list we noted that *ompK35* encodes a trimeric porin OmpK35, which is a homolog of OmpF in *Escherichia coli*. OmpK35 has been known to be associated with antibiotic resistance and virulence, along with another porin, OmpK36 [10].

**Table 3:** The top 10 genes with differentially expressed levels in wild-type, tigecycline-resistant strains (109 and 200) and tigecycline-resistant mutants (109-IR and 200-IR)

Locus_tag	Product	Fold change	RPKM		Fold change	RPKM	
			109	109-IR		200	200-IR
Up-regulated genes							
KP1_0467	DUF1471 domain-containing protein	100.860	8.556	958.148	36.543	13.677	625.192
KP1_0570	NAD(P)-dependent alcohol dehydrogenase	222.995	42.385	5534.362	185.672	29.898	4663.129
KP1_1491	MBL fold metallo-hydrolase	244.571	14.271	2441.961	131.245	17.950	2404.509
KP1_1492	RamA family antibiotic efflux transcriptional regulator	208.283	15.669	2308.962	51.519	17.314	1100.084
KP1_1863	Lipoprotein	61.709	188.337	8823.929	37.549	92.524	3182.386
KP1_2457	META domain-containing protein	36.716	157.346	3514.462	43.764	45.486	2194.189
KP1_2916	Alcohol dehydrogenase AdhP	76.506	88.219	3955.111	18.991	126.001	2476.295
KP1_3005	EamA/RhaT family transporter	25.428	4.860	127.776	49.477	3.465	261.130
KP1_3012	NAD(P)-dependent alcohol dehydrogenase	77.347	1.918	218.789	46.273	0.680	86.970
KP1_4241	Hypothetical protein	43.821	33.200	1481.113	56.380	16.806	1138.272
Down-regulated genes							
KP1_0274	Maltose ABC transporter permease MalF	− 25.282	474.168	18.524	− 6.668	25.631	4.157
KP1_0275	Maltose/maltodextrin ABC transporter substrate-binding protein MalE	− 17.645	1277.184	73.022	− 15.171	88.873	6.814
KP1_0276	maltose/maltodextrin ABC transporter ATP-binding protein MalK	− 35.866	1394.194	39.551	− 57.555	75.753	0.572
KP1_0277	Maltoporin	− 40.647	1240.421	30.963	− 11.922	42.690	3.884
KP1_0331	Murein hydrolase regulator LrgA	− 12.164	152.932	11.237	− 25.923	192.667	9.168
KP1_0332	LrgB family protein	− 22.495	159.930	5.839	− 7.642	81.850	12.069
KP1_1229	Nucleoside-specific channel-forming protein Tsx	− 3.768	829.324	242.142	− 65.419	1703.777	28.389
KP1_1929	Porin OmpK35	− 17.031	193.338	10.484	− 31.287	224.113	8.822
KP1_3535	H-type ferritin	− 5.014	240.556	45.611	− 16.395	149.821	10.912
KP1_3976	Long-chain fatty acid transporter FadL	− 15.551	152.198	8.611	− 30.253	198.279	7.948

RPKM: Read per kilobase per million

### mRNA expression of *ompK35* and *ompR*

**Fig. 2:**
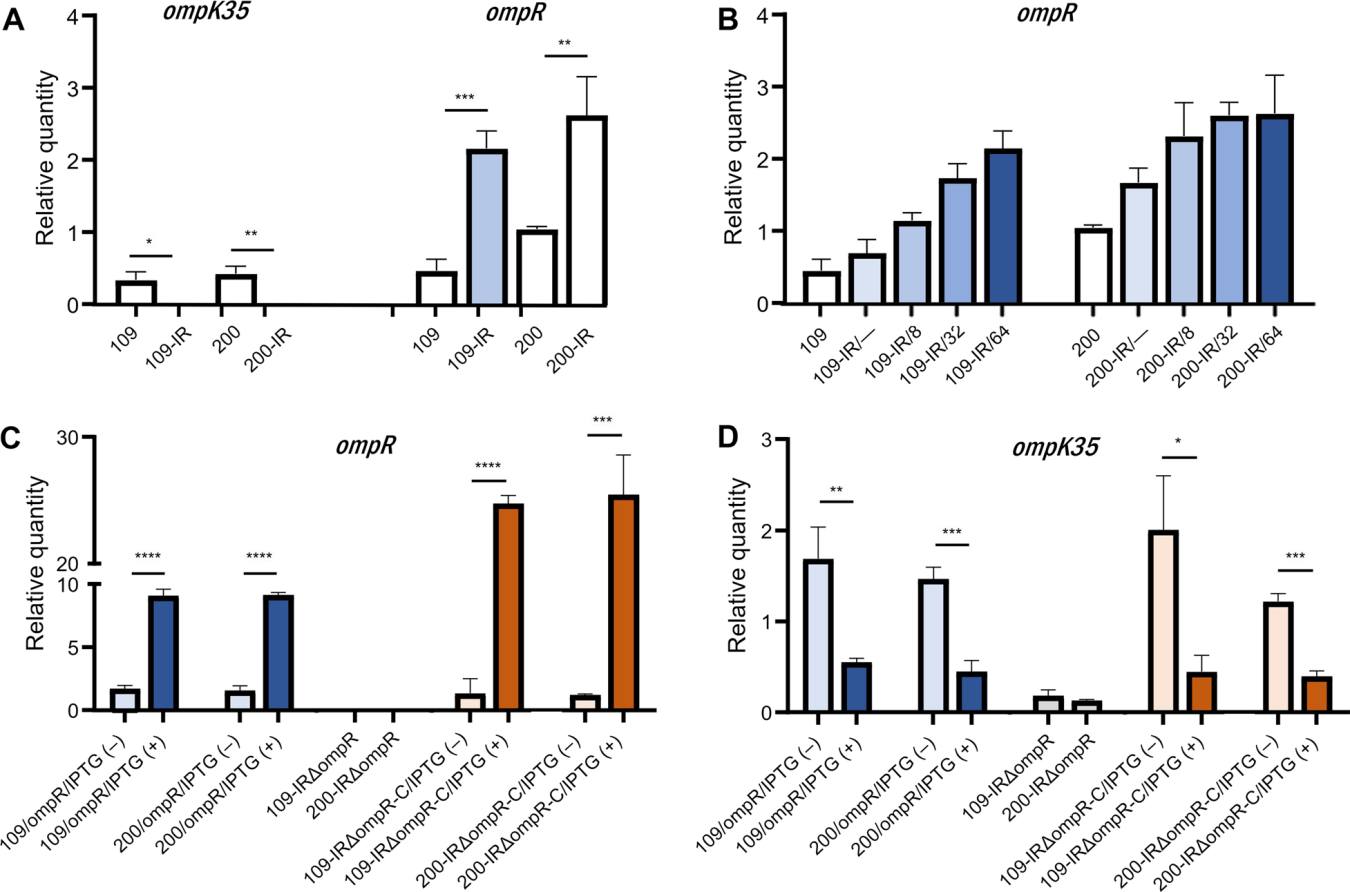
Expression levels of *ompR* and *ompK35*. **A** The expression levels of *ompK35* and *ompR* in tigecycline-susceptible, wild-type *K. pneumoniae* strains (109 and 200) and tigecycline-resistant mutants derived from wild-type strains (109-IR and 200-IR). **B** *ompR* expression levels with different tigecycline concentrations in tigecycline-resistant *K. pneumoniae* mutants (109-IR and 200-IR). **C** *ompR* expression levels in *ompR*-overexpressed mutants from wild-type strains (109/ompR and 200/ompR), *ompR*-deleted mutants from tigecycline-resistant strains (109-IRΔompR and 200-IR ΔompR), and *ompR*-complemented mutants (109-IRΔompR-C and 200-IR ΔompR-C). **D** *ompK35* expression levels in *ompR*-overexpressed mutants from wild-type strains (109/ompR and 200/ompR), *ompR*-deleted mutants from tigecycline-resistant strains (109-IRΔompR and 200-IR ΔompR), and *ompR*-complemented mutants (109-IRΔompR-C and 200-IR ΔompR-C). *P < 0.05; **P < 0.01; ***P < 0.001; ****P < 0.0001

Using qRT-PCR, we measured mRNA expression of *ompK35* and *ompR*, a repressor of *ompK35* under high osmolar environments in *E. coli* [16] in each of two wild-type strains (109 and 200) and in the tigecycline-resistant mutants 109-IR and 200-IR. As indicated by transcriptomic analysis, *ompK35* was down-regulated significantly in the tigecycline-resistant mutants 109-IR and 200-IR (*p* = 0.0108 and 0.0034, respectively) (Fig. 2A). *ompR* was up-regulated significantly in 109-IR and 200-IR, compared with their susceptible parental strains (*p*, 0.0005 and 0.0069, respectively) (Fig. 2A).

We then examined the change in *ompR* expression with increasing tigecycline concentrations in tigecycline-resistant mutants. As the concentration of tigecycline increased, the expression of *ompR* also increased gradually in both mutants (Fig. 2B). Figure 2C shows that *ompR* expression is well-regulated by the addition of IPTG.

The *ompR*-overexpressed mutants in the presence of IPTG exhibited significantly decreased expression of *ompK35* (Fig. 2D). Expression of *ompK35* increased slightly in the *ompR*-deleted mutants 109-IR∆ompR and 200-IR∆ompR, compared with the tigecycline-resistant mutants 109-IR and 200-IR (*p*, 0.0129 and 0.0011, respectively). The complementation of *ompR* and the induction of its expression in *ompR*-deleted mutants (109-IRΔompR-C and 200-IRΔompR-C) led to decreased expression of *ompK35* (Fig. 2D). These results confirmed the role of OmpR as a repressor of *ompK35*.

### Tigecycline susceptibility

The MIC for tigecycline increased eightfold in the *ompR*-overexpressed mutants 109/ompR/IPTG(+) and 200/ompR/IPTG(+), compared to those of tigecycline-susceptible, wile-type *K. pneumoniae* strains (Table 4). The tigecycline-resistant mutants exhibited very high levels of tigecycline resistance (MICs > 64 mg/L), and the MICs decreased dramatically in the *ompR* inactivated mutants 109-IR∆ompR and 200-IR∆ompR (4 and 8 mg/L, respectively). However, complementation of *ompR* did not increase the tigecycline susceptibility.

**Table 4:** Minimum inhibitory concentrations of tigecycline for wild-types and mutants

Strains	TIG MIC (mg/L)
109	1
109/ompR/IPTG(–)	2
109/ompR/IPTG(+)	8
109-IR	> 64
109-IR∆ompR	4
109-IR∆ompR-C	8
200	1
200/ompR/IPTG(–)	2
200/ompR/IPTG(+)	8
200-IR	> 64
200-IR∆ompR	8
200-IR∆ompR-C	8

TIG: tigecycline

### Phenotypic changes

**Fig. 3:**
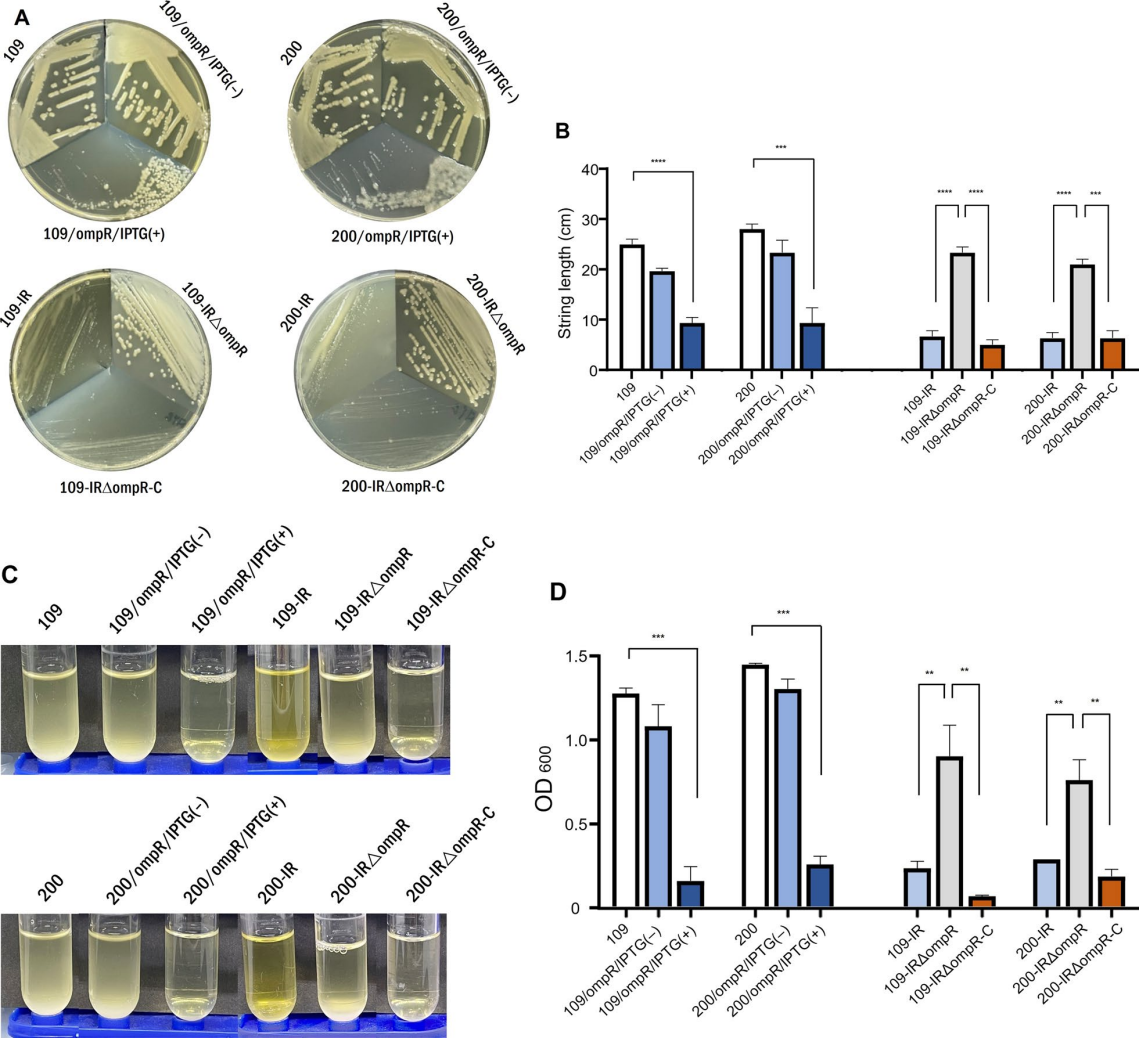
Changes in phenotypes. **A** Comparison of colony phenotypes in the strains used in this study. **B** The lengths of stretched strings in bacterial colonies. A stretched colony length of > 5 mm was defined as positive. **C**, **D** The ODs of supernatants were measured following low-speed centrifugation. The hypermucoviscous strains produced turbid supernatants. **P < 0.01; ***P < 0.001; ****P < 0.0001

To evaluate the role of *ompR* on the production of mucoviscosity in *K. pneumoniae*, we evaluated the changes in colony phenotypes in the mutants. The tigecycline-susceptible strains 109, 200, 109/ompR/IPTG(‒), and 200/ompR/IPTG(‒) displayed large and glossy colony morphologies, while the tigecycline-resistant mutants 109-IR and 200-IR exhibited small and matt colony phenotypes (Fig. 3A). The *ompR*-overexpressed mutants 109/ompR/IPTG(+) and 200/ompR/IPTG(+) also exhibited small and matt colonies, as observed with 109-IR and 200-IR (Fig. 3A). In the *ompR*-deleted mutants from the tigecycline-resistant strains 109-IR∆ompR and 200-IR∆ompR, large and glossy colony shapes were observed, compared with 109-IR and 200-IR. The small and matt colony phenotypes of 109-IR and 200-IR were restored by complementation with *ompR,* 109-IR∆ompR-C, and 200-IR∆ompR-C (Fig. 3A).

The tigecycline-susceptible wild-type strains 109 and 200 exhibited a phenotype of hypermucoviscosity, ranging from 24 to 29 mm in the string test; while the *ompR*-overexpressed mutants 109/ompR/IPTG(+) and 200/ompR/IPTG(+) exhibited significantly reduced lengths of stretched strings, an average of 9 mm for both (*P*, < 0.0001 and 0.0005, respectively) (Fig. 3B). The tigecycline-resistant mutants 109-IR and 200-IR produced string lengths of 6–8 mm; however, the string lengths of the *ompR*-deleted mutants 109-IR∆ompR and 200-IR∆ompR increased dramatically to 23 mm and 21 mm, respectively (*p*, < 0.0001 for both). Complementation with *ompR*, 109-IR∆ompR-C and 200-IR∆ompR-C, reduced the length of stretched strings to those of tigecycline-resistant mutants (Fig. 3B).

Mucoviscosity in the *K. pneumoniae* strains was determined by measuring the OD at 600 nm of the supernatants after centrifugation. The tigecycline-susceptible wild-type strains 109 and 200 produced turbid and poor sediments due to hypermucoviscosity that causes extreme stickiness (Fig. 3C). The *ompR*-overexpressed mutants 109/ompR/IPTG(+) and 200/ompR/IPTG(+), and the tigecycline-resistant mutants 109-IR and 200-IR produced clear supernatants after centrifugation, unlike the dusty appearance observed with 109, 200, 109/ompR/IPTG(‒), and 200/ompR/IPTG(‒) (Fig. 3C). The 109-IR∆ompR and 200-IR∆ompR mutants produced a turbid appearance, which reverted to a clear appearance by complementation with *ompR* (Fig. 3C).

The measurement of optical density confirmed the visual observations (Fig. 2D). The overexpression of *ompR* or the development of tigecycline resistance lowered the turbidity of supernatants significantly, that is, the mucoviscosity.

### Serum resistance

**Fig. 4:**
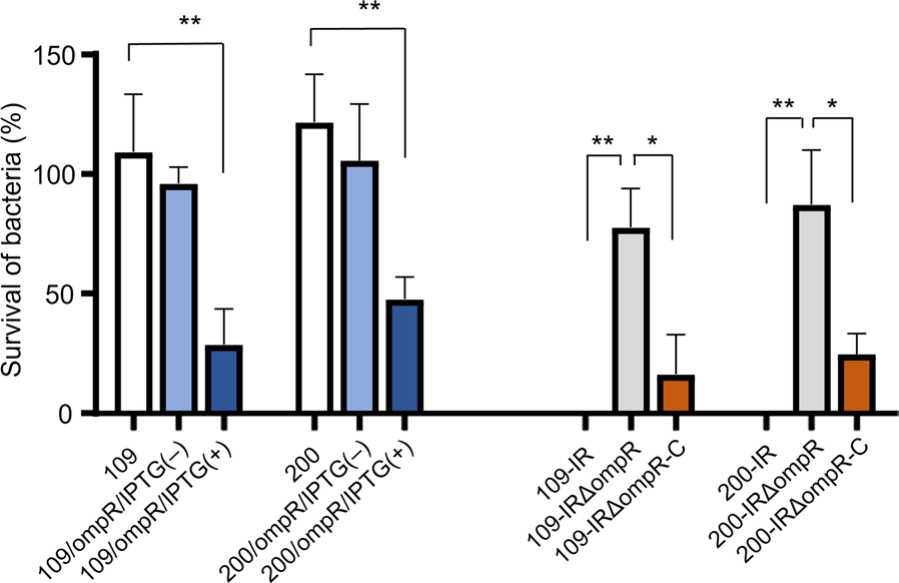
Results of serum resistance assay. The survival rates of *K. pneumoniae* strains were measured after 3 h of incubation with 75% normal human serum. Heat-inactivated serum was used as a negative control. *P < 0.05; **P < 0.01

To explore the relationship of *ompR* with virulence in *K. pneumoniae*, bacterial survival rates were evaluated against NHS (Fig. 4). The *ompR*-overexpressed mutants, 109/ompR/IPTG(+) and 200/ompR/IPTG(+), showed significantly decreased survival rates against serum, compared to the wild-type and the tigecycline-susceptible strains. The tigecycline-resistant mutants also exhibited very low survival rates against serum, which has been reported previously [12]. Deletion of *ompR* in the tigecycline-resistant mutants 109-IR∆ompR and 200-IR∆ompR increased the serum resistance (*p*, 0.0012 and 0.0026, respectively), and the survival rates of *ompR*-complemented mutants were diminished.

### Survival of *G. mellonella larva*

**Fig. 5:**
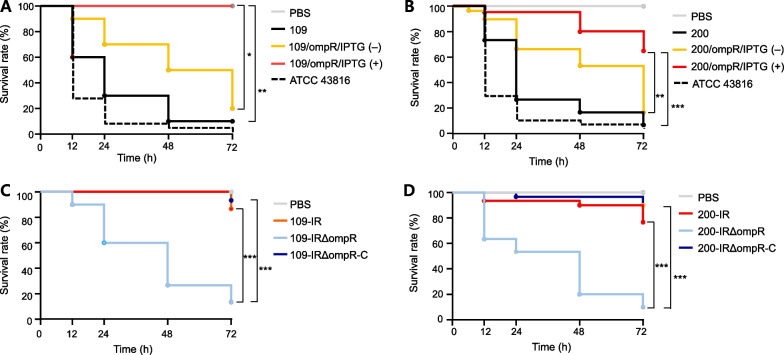
Results of *G. mellonella* larvae infection assays. **A–D** Survival curves for *G. mellonella* larvae infected with *K. pneumoniae* strains. Ten *G. mellonella* larvae were infected with each bacterial strain after adjusting with PBS to a McFarland standard of 0.5. For each strain, the results from three independent experiments were averaged. Statistical significances were represented. *P < 0.05; **P < 0.01; ***P < 0.001

Most of the *G. mellonella* larva were killed within 72 h by the strains 109 and 200, as by hypervirulent *K. pneumoniae* strain ATCC 43816 (Fig. 5A and B). The *G. mellonella* larva survived significantly longer using the *ompR*-overexpressed mutants 109/ompR/IPTG(+) and 200/ompR/IPTG(+) compared to the wild-type strains (*p*, 0.0093 and 0.0009, respectively). The tigecycline-induced resistant mutants 109-IR and 200-IR exhibited dramatically increased survival rates with G. *mellonella* larva, compared to the wild-type and the tigecycline-susceptible strains (*p*,  < 0.0001 and 0.0001, respectively) (Fig. 5A and B). Their survival rates were reduced by deletion of ompR in the tigecycline-resistant mutants and were restored by complementation with *ompR* (Fig. 5C and D).

## Discussion

Previously, we demonstrated that in vitro-induced tigecycline-resistant *K. pneumoniae* mutants exhibited a dramatic decrease in hypermucoviscosity associated with reduced capsular polysaccharide production, resulting in defects in virulence [12]. Reduced virulence with respect to serum susceptibility and survivability of *G. mellonella* has previously been reported in tigecycline-resistant *Acinetobacter baumannii* [17]. However, it is not yet known how the development of tigecycline resistance affects virulence in bacteria. In this study, we investigated which changes that occur during the development of tigecycline resistance in *K. pneumoniae* lower the virulence.

Transcriptomic analyses have confirmed that some genes that may be associated with tigecycline resistance are expressed differentially in two tigecycline-resistant mutants, for example, *ramA* [18]. We detected decreased expression of a porin, OmpK35, in both tigecycline-resistant mutants. In *K. pneumoniae*, OmpK35 is a homolog of the *E. coli* OmpF porin [19]. It is responsible for rapid influx of β-lactams including third generation cephalosporins and carbapenems in *K. pneumoniae* clinical isolates [20, 21]. Thus, a deficiency or a defect in the porin results in resistance to cephalosporins and carbapenems. Recently, decreased expression of OmpK35 was also identified in tigecycline-resistant *K. pneumoniae* strains [11]. In addition to antibiotic resistance, decreased virulence has been reported in *ompK35*-deficient *K. pneumoniae* and *ompF*-deficient *E. coli* mutants [10, 22]. Since OmpK35 and OmpK36 are known to be regulated by a two-component regulatory system, OmpR-EnvZ-sensing osmotic signals [23, 24], we explored the effects of OmpR on the phenotypic changes that lead to virulence in the tigecycline-resistant mutants.

First, we confirmed that tigecycline-resistant mutants overexpressed *ompR* and that *ompK35* expression is regulated negatively by *ompR*. In addition, the expression of *ompR* increased with increasing concentrations of tigecycline. That is, OmpR might sensor the tigecycline, which may act as an osmolarity factor.

Exposure to tigecycline produced a change in the phenotypes in hypervirulent *K. pneumoniae* strains. While tigecycline-susceptible and hypervirulent strains produced large, glossy, and mucoid colonies, the colonies of tigecycline-resistant mutants generated small, matt, and non-mucoid which may lead to the reduced virulence. Similar phenotypic changes were also observed in *ompR*-overexpressed mutants. In addition, after the deletion of *ompR*, tigecycline-resistant mutants were restored like phenotype of the tigecycline-susceptible *K. pneumoniae* strains. This implies that the overexpression of OmpR induced by tigecycline exposure can be responsible for the phenotypic changes in the hypervirulent *K. pneumoniae* stains.

The phenotype changes in tigecycline-induced resistance and *ompR* overexpression were clearly associated with decreased virulence judged from the survival of NHS and *G. mellonella* larvae. The altered susceptibility to tigecycline caused by the overexpression or deletion of *ompR* may be associated with OmpK35 expression, which is regulated by OmpR. The smaller change caused by complementation with *ompR* may indicate that the influence of OmpR on the tigecycline susceptibility is indirect.

Our study have some limitations. First, only a few strains were studied, limiting the generalization of the results. Second, it has not been revealed what features of tigecycline affect the expression of *ompR* and *compK35*. Nor have we found out why other antibiotics do not have these phenomena. CPS is known to be synthesized in different ways in *K. pneumoniae* K1 serotype, and thus would be further confirmed by various assays, for example, capsule stain and Periodic Acid Schiff stain. In addition, OmpK35 is a constituent of channel for extracellular polysaccharide (EPS) as well as CPS. As EPS may also be crucial for virulence, presence or amount of EPS should be compared between tigecycline-resistant and -susceptible strains.

Based on the findings in this study, we speculated that the cause of the increased resistance to tigecycline and reduced virulence in hypermucoviscosity and hypervirulent *K. pneumoniae* strains attributed to the overexpression *ramA* by exposure to tigecycline [7, 12]. Tigecycline may simultaneously stimulate OmpR. Overexpressed OmpR binds *ompK35*, and acts as a repressor. Down-regulated OmpK35 caused by OmpR resulted in reduced virulence in hypermucoviscous and hypervirulent *K. pneumoniae* strains.

## Conclusions

In the present study, we demonstrated that *ompR* expression is regulated by exposure to tigecycline, thereby affecting the virulence associated phenotypes in *K. pneumoniae* strains. The reduced virulence in tigecycline-resistant mutants is probably an accompanying action of OmpK35, which is negatively regulated by OmpR.

## Data Availability

All materials are available by the corresponding author.

## Abbreviations

CPS: Capsular polysaccharide
ST: Sequence type
LB: Luria–bertani
IPTG: Isopropyl-β-d-thiogalactopyranoside
RPKM: Reads per kilobase per million
Km: Kanamycin resistance
MIC: Minimum inhibitory concentration
OD: Optical density
NHS: Normal human serum
PBS: Phosphate buffered saline
qRT-PCR: Quantitative real-time PCR
